# Evaluation of the Shape Symmetry of Bilateral Normal Corneas in a Chinese Population

**DOI:** 10.1371/journal.pone.0073412

**Published:** 2013-08-29

**Authors:** Fangjun Bao, Hao Chen, Ye Yu, Jiguo Yu, Shi Zhou, Jing Wang, QinMei Wang, Ahmed Elsheikh

**Affiliations:** 1 The Affiliated Eye Hospital of WenZhou Medical College, Wenzhou, China; 2 School of Engineering, University of Liverpool, Liverpool, United Kingdom; 3 National Institute for Health Research, Biomedical Research Centre at Moorfields Eye Hospital, NHS Foundation Trust, and UCL Institute of Ophthalmology, United Kingdom; Cedars-Sinai Medical Center, United States of America

## Abstract

**Purpose:**

To investigate the bilateral symmetry of the global corneal topography in normal corneas with a wide range of curvature, astigmatism and thickness values

**Design:**

Cross-Sectional Study

**Methods:**

Topography images were recorded for the anterior and posterior surfaces of 342 participants using a Pentacam. Elevation data were fitted to a general quadratic model that considered both translational and rotational displacements. Comparisons between fellow corneas of estimates of corneal shape parameters (elevation, radius in two main directions, R_x_ and R_y_, and corresponding shape factors, Q_x_ and Q_y_) and corneal position parameters (translational displacements: x_0_, y_0_ and z_0_, and rotational displacements: α, β and γ) were statistically analyzed.

**Results:**

The general quadratic model provided average RMS of fit errors with the topography data of 1.7±0.6 µm and 5.7±1.3 µm in anterior and posterior corneal surfaces. The comparisons showed highly significant bilateral correlations with the differences between fellow corneas in R_x_, R_y_, Q_x_ and Q_y_ of anterior and posterior surfaces remaining insignificantly different from zero. Bilateral differences in elevation measurements at randomly-selected points in both corneal central and peripheral areas indicated strong mirror symmetry between fellow corneas. The mean geometric center (x_0_, y_0_, z_0_) of both right and left corneas was located on the temporal side and inferior-temporal side of the apex in anterior and posterior topography map, respectively. Rotational displacement angle α along X axis had similar distributions in bilateral corneas, while rotation angle β along Y axis showed both eyes tilting towards the nasal side. Further, rotation angle γ along Z axis, which is related to corneal astigmatism, showed clear mirror symmetry.

**Conclusions:**

Analysis of corneal topography demonstrated strong and statistically-significant mirror symmetry between bilateral corneas. This characteristic could help in detection of pathological abnormalities, disease diagnosis, measurement validation and surgery planning.

## Introduction

Characterization of corneal topography using computer-assisted topography systems [1] is critical for the assessment of vision quality and in several clinical applications including fitting of contact lenses [2],[3], diagnosis and management of keratoconus [4],[5] planning of refractive surgery [6],[7] and construction of corneal numerical models [8],[9].

The external human body has a highly bilateral symmetrical structure relative to the mid-sagittal plane, and this feature has been helpful in detection of pathological abnormalities, disease diagnosis and measurement validation [10], [11], [12]. With reference to the cornea, bilateral symmetry was recognized in the late 19^th^ century when Mach noted that symmetry was more easily recognized about the vertical mid-sagittal plane than about other plane orientations [13]. More recently, a high degree of bilateral corneal symmetry has been observed in measurements of higher-order corneal aberrations [14], the relation of astigmatism axis orientation [15], diameter of myopic corneas [16] and location of thinnest corneal point along vertical midline [17], in addition to other localized features including central corneal thickness, curvature and elevation [18]. In addition to the assessment of some of these earlier observations on bilateral symmetry, this study investigates symmetry in the global shape of normal corneas with a wide range of curvature, refractive error, astigmatism and thickness values.

With reference to a clinical dataset collected at the Wenzhou Medical College, the study presents an assessment of the bilateral symmetry of corneal shape in 342 subjects and includes comparisons of radius of curvature, shape factor, elevation and both translational and rotational displacements.

## Materials and Methods

342 healthy subjects (202 male and 140 female) aged between 17 and 45 years (mean age 24.42 ± 5.40 years) were recruited from corneal refractive surgery patients and medical trainees of the Eye Hospital of Wenzhou Medical College, China. The study followed the tenets of the Declaration of Helsinki and was approved by the Scientific Committee of the Eye Hospital. Signed informed consent that allowed use of the data for research was obtained from all participants. The inclusion criteria were corneal astigmatism less than 3.00D, bilateral difference of mean corneal curvature below 3.00D, no previous ocular surgery, no history of trauma and no ocular disease, and no contact lens wear for at least two and four weeks before topography measurement for soft contact lens and gas permeable contact lens (RGP) wearers, respectively. Eyes that did not meet these criteria were excluded.

The study parameters included refractive error (RE), mean corneal curvature (K_m_, the mean of curvature in horizontal and vertical directions), corneal astigmatism (CA, the difference between horizontal and vertical corneal curvature), central corneal thickness (CCT), minimum corneal thickness (MCT), and corneal elevation data of anterior and posterior surfaces. RE was measured with a phoroptor (RT-2100, Nidek Inc, Gamagori, Japan) and converted to spherical equivalent, SE. K_m_, CA, CCT, MCT and corneal elevation were provided by a Pentacam (OCULUS Optikgerate GmbH, Wetzlar, Germany). Central and minimum corneal thickness variation ratio (CMVR), a parameter used to describe the variation in thickness, was calculated as the ratio between CCT and MCT. Room lights were switched off during data acquisition. Subjects were positioned with a headrest and instructed to fixate on the internal fixation lamp. They were asked to open both eyes and blink just before each measurement was taken. After each acquisition, the device was moved back and realigned for the next scan. The measurement sequence was OD-OS, and continued until three scans with an instrument-generated quality factor of at least 95% and 90% were obtained for the anterior and posterior surfaces, respectively. All measurements were taken by the same trained examiner (JC).

**Figure 1:**
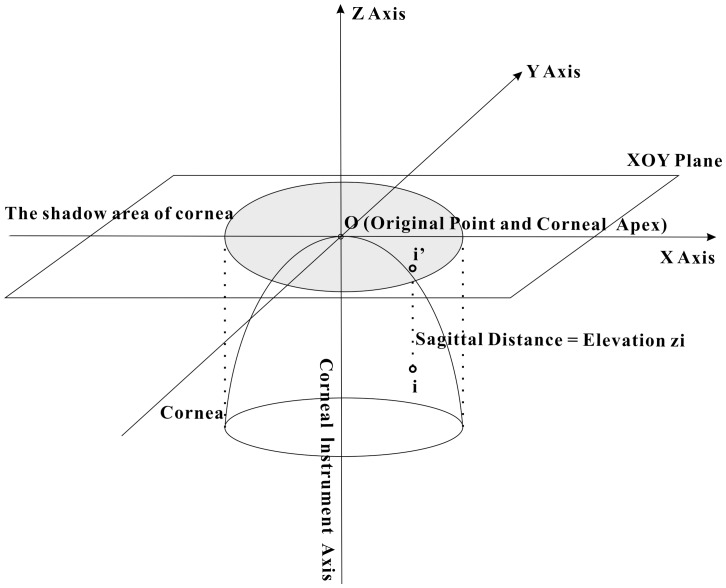
Elevation data obtained from the Pentacam. O, denoting the apex of anterior or posterior corneal surface, is the origin point of the Cartesian coordinate system, XOY is the plane that passes through X and Y axes and Point O. Elevation zi is the sagittal distance between a general point, i, and the XOY plane.

**Figure 2:**
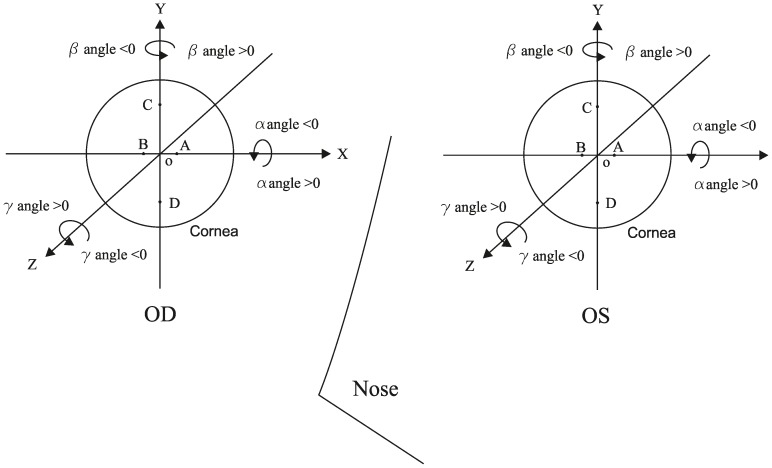
The Cartesian coordinate system of bilateral corneas. Points A to D were chosen to test instrument measurement repeatability and analyze interocular symmetry. Rotation angles α, β and γ could be positive or negative according to right hand spiral law.

The elevation maps of each anterior and posterior surface were exported in the form of up to 6361 elevation data points representing a middle circle of 9 mm diameter. A Cartesian coordinate system was employed with the X, Y and Z-axes in the horizontal (OD: temporal-nasal; OS: nasal-temporal), vertical (inferior-superior) and sagittal (posterior-anterior) directions, respectively. The data consisted of elevation z_i_ at each point i on either the anterior or posterior corneal surface with Cartesian coordinates (x_i_, y_i_). z_i_ was defined as the sagittal distance between point i and an XOY plane passing through the origin point O (the apex of anterior or posterior corneal surface), at which the instrument axis intercepts the cornea (Fig. 1). The data at four points including two in the central area: A (1, 0, z_A_) and B (−1, 0, z_B_), and two in the periphery: C (0, 3, z_C_) and D (0, −3, z_D_), were further used to test repeatability and symmetry of measurements (Fig. 2).

The study used the general quadratic model to estimate values of the radii of curvature R_x_ and R_y_ and shape factors Q_x_ and Q_y_ based on the coordinate data (x, y, z):
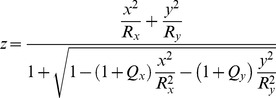



In using the elevation data, the model considered a change from the instrument's coordinate system to the intrinsic corneal coordinate system by applying the necessary 3D translations and rotations. The intrinsic corneal coordinates (x, y, z) of the surface are given in matrix-vector formulation by
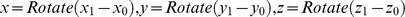
where (x_1_, y_1_, z_1_) are column vectors representing coordinates in the instrument system, (x_0_, y_0_, z_0_) scalars representing the translational displacements, and hence the new coordinates of the centre of the quadratic, and Rotate is a 3D rotation matrix that complies with the Euler rotation theorem [19], and can be given as a composition of angular rotations about the three main axes: 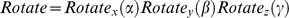
where



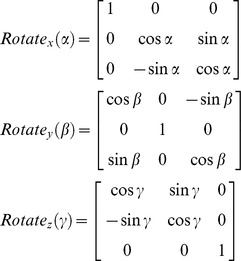
and α, β, γ represent the angular rotations about the three main axes (Fig. 2). In this study, the quadratic equation was fitted to the elevation data of each topography map and the root mean squared error of fit, RMS (
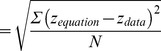
, where N is the number of data points), was reduced to a minimum using the least squares method and leading to the best values of the shape parameters (R_x_, R_y_, Q_x_, Q_y_), the translations (x_0_, y_0_, z_0_) and the Euler angles of rotation (α, β, γ).

Commercial software SPSS Statistics (version 20.0, IBM, Inc.) was utilized for all statistical analyses. The normality of anatomic data distributions was confirmed with the Kolmogorov-Smirnov test, and parametric statistical tests were used for data analyses. Repeatability of the three topography measurements and the fitting results for each corneal surface was assessed using the within-subject standard deviation (Sw), intraoberver precision (Pr), within-subject coefficient of variation (CV) and intraclass correlation coefficient (ICC). The mean fitting data of 3 measurements of each cornea was used to evaluate the agreement between fellow corneas, and paired-samples t test was chosen to compare the differences between bilateral corneas. Bilateral differences of elevation measurement at points A-D were tested by Mann-Whitney U-test. The correlation analyses were assessed using the Pearson's or Spearman linear correlation factor according to a normal distribution test. The agreement between bilateral corneas was investigated with the Bland- Altman Plots.

## Results

There was a wide range of RE (−13.25 to 5.13 D), K_m_ (39.80 to 47.33 D), CA (0.04 to 2.98 D) and CCT (460.67 to 619.33 µm), MCT (455.00 to 617.33 µm), CMVR (1.00 to 1.02). The differences in RE, K_m_, CA and CCT between fellow eyes were not significant unlike the differences in MCT and CMVR (Table 1). Table 2 shows the Sw, Pr, CV and ICC values for the three consecutive topography measurements of anterior and posterior elevation maps obtained with the Pentacam. The repeatability of elevation measurement was excellent. The CV values did not exceed 0.8% and 1.7%, and the ICC values were all greater than 0.95 and 0.90 for anterior and posterior surface measurements, respectively.

**Table 1:** Values of refractive error, corneal curvature, astigmatism and central thickness.

Ocular Parameters	OD	OS	Interocular Differences (OD-OS)	OD Vs. OS P Value	Interocular correlation, r
SE, D	−4.69 ± 2.68 (−13.25 – 5.13)	−4.63 ± 2.60 (−12.63 – 4.50)	−0.07 ± 0.83 (−3.75 – 3.88)	0.054	0.95 (P<0.01)
K_m_, D	43.39 ± 1.41 (39.90 – 47.13)	43.40 ± 1.41 (39.80 – 47.33)	−0.01 ± 0.25 (−0.78 – 0.67)	0.682	0.98 (P<0.01)
CA, D	1.03 ± 0.53 (0.05 – 2.98)	1.06 ± 0.55 (0.04 – 2.88)	−0.03 ± 0.32 (−0.77 – 0.79)	0.141	0.80 (P<0.01)
CCT, µm	541.17 ± 28.40 (467.33 – 619.33)	540.52 ± 28.52 (460.67 – 616.67)	0.65 ± 6.36 (−19.67 – 18.67)	0.059	0.98 (P<0.01)
MCT, µm	538.39 ± 28.47 (465.33 – 617.33)	537.58 ± 28.61 (455.00 – 612.33)	0.81 ± 6.38 (−18.33 – 19.33)	0.019	0.98 (P<0.01)
CMVR	1.01 ± 0.00 (1.00 – 1.02)	1.01 ± 0.00 (1.00 – 1.02)	−0.0003 ± 0.0026 (−0.0121 – 0.0067)	0.032	0.57 (P<0.01)

SE = refractive error and converted to spherical equivalent, K_m_ = mean corneal curvature (the mean of corneal curvature in horizontal and vertical axis); CA = corneal astigmatism (the difference between corneal curvature in horizontal and vertical axis); CCT = central corneal thickness, MCT = minimum corneal thickness; CMVR = the ratio between CCT and MCT.

**Table 2:** Intraobserver repeatability outcomes for elevation measurements obtained at four points on corneal surface.

Corneal Surface	Point	Mean elevation ±SD, µm	Sw, µm	Pr, µm	CV,%	ICC(95%CI)
Anterior	A_OD_	63.70±2.09	0.47	0.91	0.73	0.951(0.941–0.959)
	B_OD_	64.55±2.12	0.44	0.86	0.68	0.958(0.950–0.965)
	C_OD_	605.61±23.56	1.62	3.18	0.27	0.995(0.994–0.996)
	D_OD_	606.77±22.72	1.48	2.9	0.24	0.996(0.995–0.996)
	A_OS_	64.42±2.21	0.47	0.91	0.72	0.956(0.947–0.963)
	B_OS_	63.74±2.08	0.46	0.91	0.73	0.951(0.941–0.959)
	C_OS_	605.11±23.34	1.93	3.79	0.32	0.993(0.992–0.994)
	D_OS_	608.31±22.63	1.91	3.74	0.31	0.993(0.992–0.994)
Posterior	A_OD_	79.59±4.16	1.29	2.53	1.65	0.904(0.886–0.919)
	B_OD_	71.37±3.59	1.13	2.22	1.61	0.901(0.882–0.917)
	C_OD_	798.60±37.29	6.08	11.92	0.76	0.973(0.968–0.978)
	D_OD_	758.70±35.14	5.62	11.02	0.74	0.974(0.969–0.979)
	A_OS_	71.27±3.84	1.04	2.05	1.48	0.926(0.912–0.938)
	B_OS_	78.68±4.43	1.27	2.48	1.61	0.918(0.903–0.932)
	C_OS_	797.68±37.36	6.17	12.1	0.77	0.973(0.967–0.977)
	D_OS_	758.81±34.86	5.94	11.64	0.78	0.971(0.965–0.976)

Sw = within-subject standard deviation; Pr = intraobserver precision; CV = coefficient of variation; ICC = intraclass correlation coefficient; A_OD_ is point A on the right cornea.

The quality of fit with the general quadratic model was acceptable with RMS of error of 1.73±0.57 µm (range 0.69–5.78 µm) and 5.70±1.33 µm (range 2.61–11.77 µm) in anterior and posterior surfaces, respectively, and corresponding R^2^ of 0.9993±0.0009 (range 0.9917–1.0000) and 0.9988±0.0016 (range 0.9882–1.0000). The fitting results of anterior corneal surfaces were consistently better than those of posterior surfaces.

A test of bilateral symmetry concentrated on the elevation data at points A_OD_ and B_OS_, and similarly A_OS_ and B_OD_, whose locations are illustrated in Fig. 2. Table 3 shows that the elevation differences between points positioned with bilateral mirror symmetry (A_OD_ vs B_OS_ and A_OS_ vs B_OD_) had lower values than those between similarly located points, i.e. A_OD_ vs A_OS_ and B_OD_ vs B_OS_.

**Table 3:** Bilateral differences of elevation measurements at two symmetrically positioned points on corneal surface.

Corneal surface	Elevation difference			Elevation difference		
	Mean± SD, µm		Comparison	Mean± SD, µm		Comparison
	A_OD_ - Aos	A_OD_ - B_OS_		B_OD_ - B_OS_	B_OD_ - A_OS_	
Anterior	−0.72±0.90	−0.04±0.72	Z = −9.64 (P = 0.00)	0.81±0.88	0.13±0.63	Z = −10.41 (p = 0.00)
Posterior	8.32±4.74	0.92±2.45	Z = −19.06 (p = 0.00)	−7.31±4.54	0.09±2.73	Z = −19.34 (P = 0.00)

A_OD_ = Point A on right cornea, B_OD_ = Point B on right cornea, A_OS_ = Point A on left cornea, B_OS_ = Point B on left cornea, Z = output of Mann-Whitney U-test.

Table 4 presents the mean, SD, range and interocular differences in measurements of R_x_, R_y_, Q_x_, Q_y_ as obtained from the general quadratic model for both anterior and posterior surfaces of right and left eyes. The comparisons show highly significant correlations between fellow corneas with correlation coefficients higher than 0.95 (P<0.001) and 0.75 (P<0.001) for R and Q, respectively. However, while the differences between fellow corneas in R_x_, R_y_, Q_x_ and Q_y_ of anterior surfaces were not statistically significant, analysis of posterior maps showed statistically significant differences in R_y_, Q_x_ and Q_y_ but not R_x_.

**Table 4:** Differences between corneal shape parameters determined for the right and left eyes.

Corneal surface	Corneal shape parameters	OD	OS	Interocular Difference	OD Vs. OS P Value	Interocular correlation, r
Anterior	R_x_	7.81±0.25	7.81±0.26	−0.002±0.053	0.40	0.98 (P<0.01)
	R_y_	7.62±0.28	7.62±0.27	0.005±0.062	0.14	0.98 (P<0.01)
	Q_x_	−0.27±0.09	−0.27±0.08	0.001±0.055	0.88	0.84 (P<0.01)
	Q_y_	−0.28±0.12	−0.27±0.12	−0.004±0.069	0.21	0.82 (P<0.01)
Posterior	R_x_	6.35±0.25	6.35±0.26	0.001±0.076	0.86	0.96 (P<0.01)
	R_y_	5.96±0.26	5.95±0.25	0.012±0.077	0.01	0.96 (P<0.01)
	Q_x_	−0.15±0.11	−0.16±0.10	0.012±0.066	0.00	0.78 (P<0.01)
	Q_y_	−0.42±0.14	−0.43±0.14	0.009±0.075	0.02	0.85 (P<0.01)

**Figure 3:**
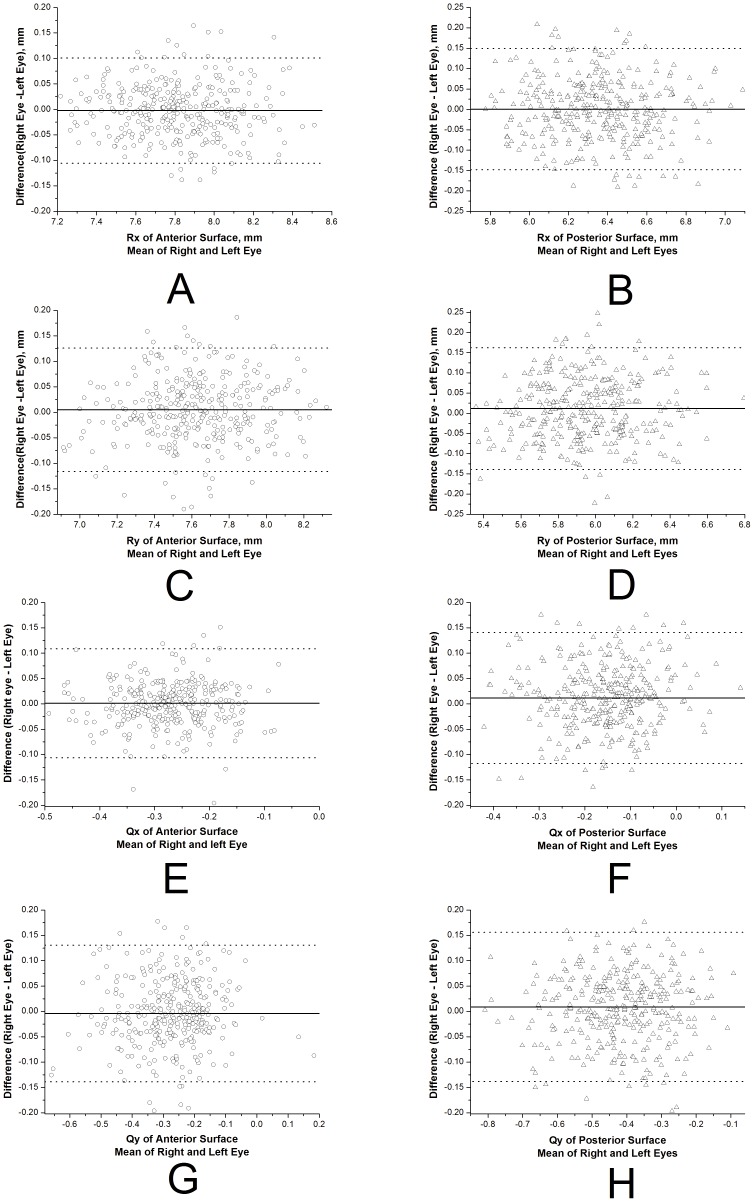
Bland-Altman plots for analysis of the agreement between bilateral eyes in R_x_, R_y_, Q_x_ and Q_y_ of anterior (A, C, E, G) and posterior surfaces (B, D, F, H).

Additionally, the mean differences between fellow corneas in R_x_, R_y_, Q_x_ and Q_y_ of anterior and posterior surfaces were not significantly different from zero as shown in the Bland-Altman plots of Fig. 3. Finally, in terms of the agreement between bilateral corneas, R_x_ and R_y_ of anterior and posterior surfaces showed a narrow 95% LoA, which implies good agreement. However, the 95% LoA was relatively broad for Q_x_ and Q_y_, indicating moderate agreement.

**Figure 4:**
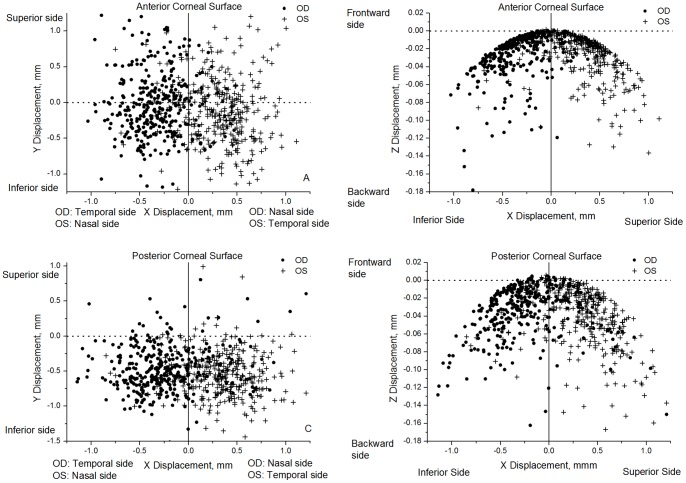
Values of X, Y and Z translation displacements estimated for all 342 participants. With the right eye viewed from the measuring instrument the coordinate convention is positive X toward the nasal side, while for the left eye positive X is towards the temporal side. In both eyes, the coordinate convention has positive Y towards the superior side and positive Z towards the frontward side.

The results further include the coordinates of the geometric center of corneal surface (expressed by translational displacements x_0_, y_0_, z_0_) depicted in Fig. 4 and Table 5. The mean geometric center of both the right and left corneas are located on the temporal side and inferior-temporal side of the apex in anterior (Fig. 4A) and posterior surfaces (Fig. 4C), respectively. These results provide further evidence of mirror symmetry relative to the median vertical, YZ, plane (interocular correlation of x_0_, r = −0.72 and r = −0.52 for anterior and posterior surfaces, respectively). Similar results were obtained for the Z translational displacement (z_0_), which were relatively small and similar among fellow corneas for both anterior (Fig. 4B) and posterior surfaces (Fig. 4D).

**Table 5:** Translational and rotational displacement results of general quadratic model.

Corneal surface	Displacement	OD	OS	Interocular correlation, r
Anterior	x_0_, mm	−0.33±0.28	0.34±0.30	−0.72 (P<0.01)
	y_0_, mm	−0.02±0.47	−0.11±0.49	0.81 (P<0.01)
	z_0_, mm	−0.03±0.03	−0.03±0.03	0.65 (P<0.01)
	α, degree	−0.13±3.54	−0.83±3.68	0.81 (P<0.01)
	β, degree	2.42±2.06	−2.53±2.23	−0.72 (P<0.01)
	γ, degree	−2.70±21.26	2.77±19.05	−0.47 (P<0.01)
Posterior	x_0_, mm	−0.24±0.38	0.32±0.34	−0.52 (P<0.01)
	y_0_, mm	−0.47±0.32	−0.54±0.31	0.71 (P<0.01)
	z_0_, mm	−0.04±0.03	−0.04±0.03	0.57 (P<0.01)
	α, degree	−3.95±2.96	−4.64±2.91	0.72 (P<0.01)
	β, degree	2.00±3.37	−2.66±2.95	−0.52 (P<0.01)
	γ, degree	−2.69±11.92	4.44±12.20	−0.44 (P<0.01)

**Figure 5:**
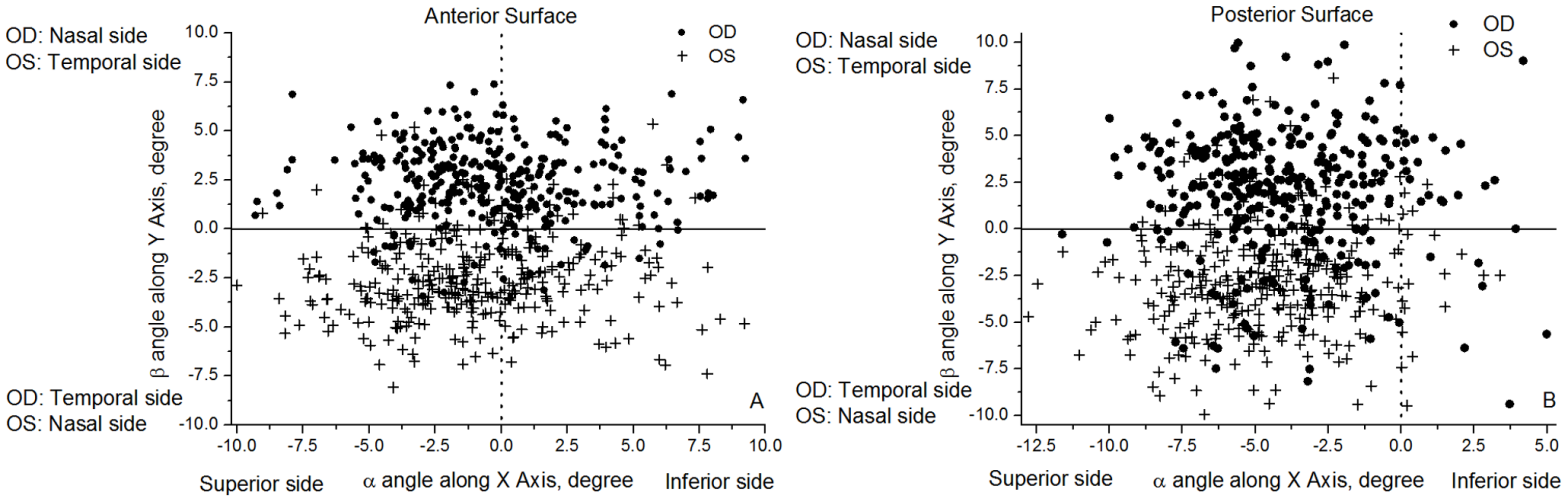
Values of α, β rotation angles estimated for all 342 participants. With the right eye viewed from the measuring instrument the coordinate convention is positive β towards the nasal side, while for the left eye, positive X is towards the temporal side. In both eyes, the coordinate convention has positive α toward the inferior side.

**Figure 6:**
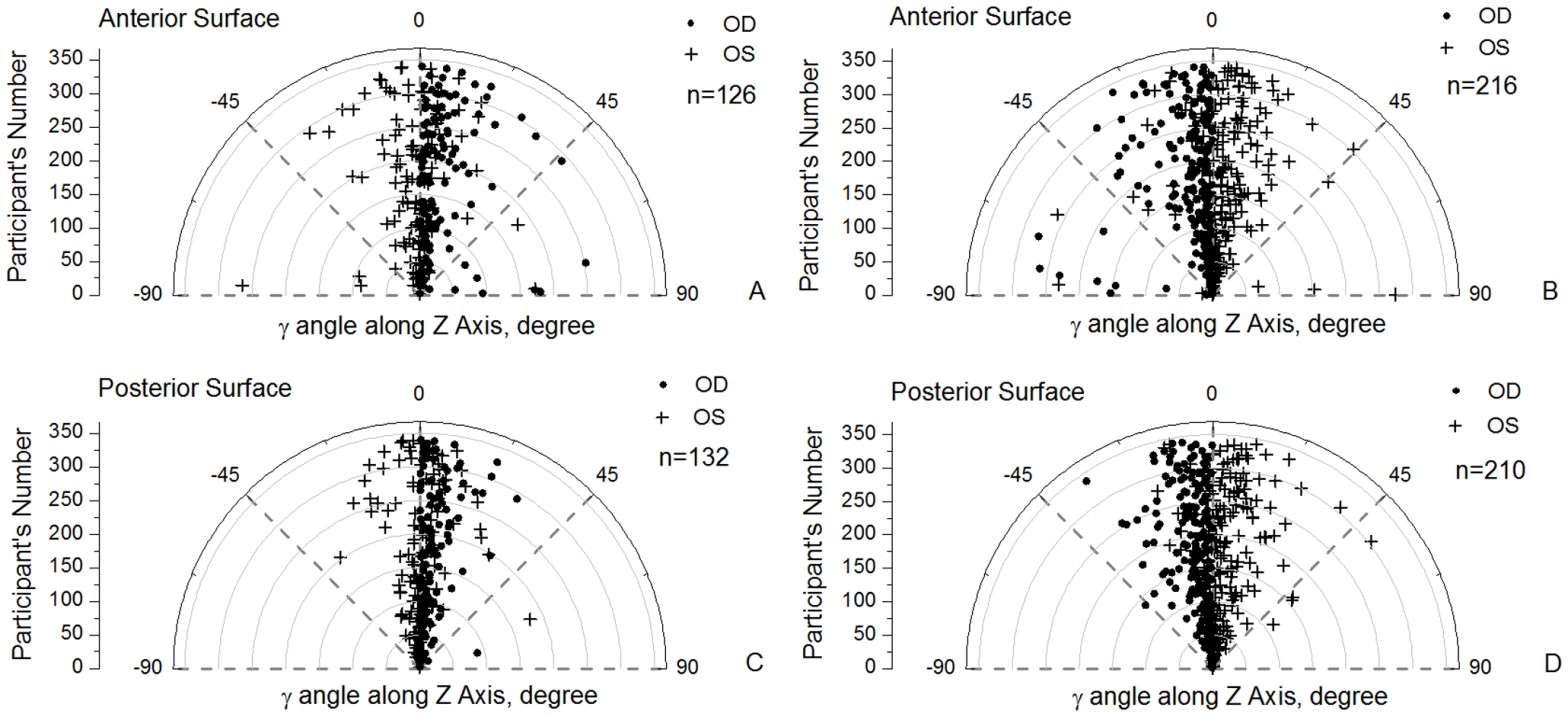
Values of γ rotational angles estimated for all 342 participants. For clarity of presentation, participants were split into two groups according to whether γ of right corneas was greater than 0 (A, C) or less than 0 (B, D).

The analysis was extended to the angles of rotational displacement between the geometric and keratometric axes of corneal surfaces as shown in Figs. 5 and 6 and Table 5. Angle α had similar distributions in bilateral corneas (r = 0.81 and r = 0.72 for anterior and posterior corneal surfaces) with the same angle signs in fellow corneas making the correlation parameters positive (Fig. 5). Similar results were obtained for angle β although in this case the values for right and left corneas had opposite signs (both rotated towards the nasal side), and therefore the correlation parameters were negative: r = −0.72 and r = −0.52 for anterior and posterior surfaces, Fig. 5. Furthermore, angle γ, which was related to corneal astigmatism, also had opposite signs for right and left corneas with the absolute values indicating a high degree of mirror symmetry relative to the median vertical plane YZ in Fig. 6 (r = −0.47 and r = −0.44 for anterior and posterior surfaces).

## Discussion

Most recent studies on bilateral corneal symmetry concentrated on the cornea's localized features such as higher-order aberrations, astigmatism axis orientation, diameter of myopic corneas, central thickness and posterior elevation at fixation point [13], [14], [15], [16], [17], [18]. Only one study [19] assessed the bilateral symmetry of overall corneal topography using anterior surface maps of 53 participants and reported similar findings to the current study. With help of mathematic analysis, this study aims to complement earlier research through investigating the global symmetry of anterior and posterior surfaces of normal corneas. Based on the topographic analysis of 342 pairs of eyes of healthy participants with a wide range of refractive error, corneal curvature, astigmatism and thickness, the study demonstrated considerable and statistically significant mirror symmetry between fellow corneas.

The accuracy of topography measurements was ensured by discarding maps of anterior and posterior surfaces with quality factors below 95% and 90%, respectively, using the same trained examiner to acquire all data, and repeating measurements at least three times. The intra-observer repeatability of elevation measurements was evaluated and found to confirm the consistency and repeatability of measurements. The intra-oberver precision (Pr) remained was bellow 3.80 µm and 12.20 µm in all anterior and posterior surfaces, respectively. The elevation data obtained at points A and B (in the cornea's central area) and points C and D (in the peripheral area) were also analyzed. The Sw and Pr values, practical indexes of measurement error, were approximately 4 times larger in the peripheral area and showed a higher resolution in the central area. On the other hand, the CV and ICC values were obtained while assessing the measurement error against the subject mean and normalizing its value [20], [21]. An order of magnitude difference was obtained in the mean elevation data between the central and peripheral areas with higher CV and lower ICC results in the former region, thus indicating better repeatability in the peripheral area (Table 2).

A high degree of interocular corneal shape symmetry was observed in the values of corneal radius (R), shape factor (Q) and z-elevations. Although there was a large variation in corneal curvature (39.80 to 47.33D) within the group of participants, R, Q and elevation values remained similar in the anterior maps of fellow eyes. However, the differences between fellow eyes in the posterior R_y,_ Q_x_ and Q_y_, but not Rx, were statistically significant, which could be caused by the distortion in tracing posterior maps caused by the anterior surface and the considerable variation in corneal thickness (the range of bilateral difference of CCT was −19.67 to 18.67 µm), and that was supported by the fact that statistically significant correlation could be found between the bilateral differences in CMVR and the posterior Ry, Qx and Qy (r = 0.224, P<0.000; r = −0.175, P<0.000; r = 0.156, P<0.000).

**Figure 7:**
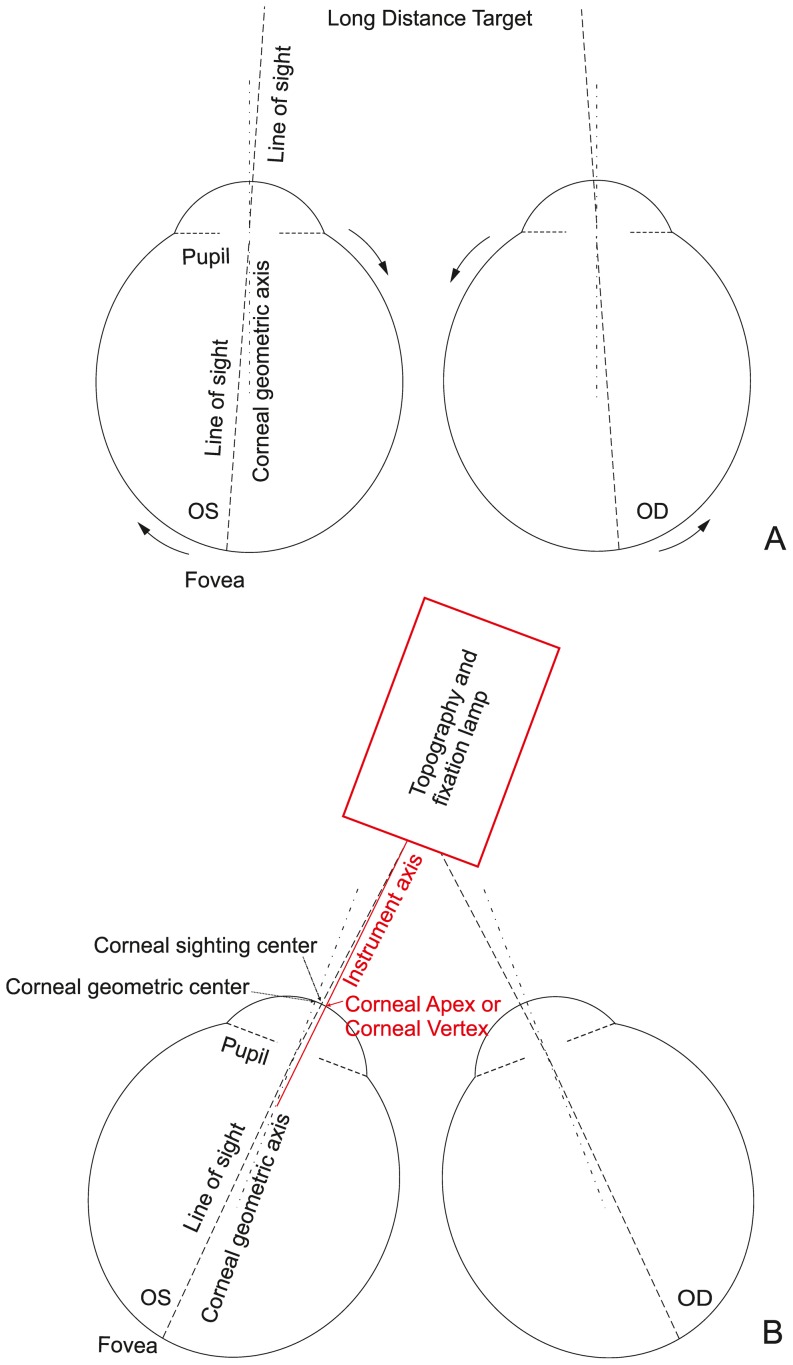
Convergence procedure. Bilateral eyes fixed on a long-distance target (A). When eyes are fixed on a near-distance target during measurement, bilateral eyes turn inward (B). Corneal geometric axis turns towards the nasal side and corneal apex provided by the instrument is located on the nasal side of the corneal geometric center.

The study also evaluated agreement of corneal geometric center location between fellow eyes, and high mirror symmetry has been found in both anterior and posterior surfaces. The mean geometric centers of anterior surfaces in right and left corneas were located on the temporal side of the apex and the corresponding mean geometric axes involved rotation towards the nasal side of the keratometric axis of cornea. On the other hand, the mean geometric centers of posterior surfaces were on the inferior-temporal side of apex and the axes rotated towards the superior-nasal side. These findings are likely to be related to the instruction given to participants to fix their gaze on the target lamp in near distance during topography measurement, which would cause the convergence that makes both eyes turn inward (Fig. 7). The apex in Pentacam topography maps, defined as the point at which the mathematical gradient reduces to zero, coincides with corneal vertex where the instrument axis intersects the cornea [22]. In this study corneal vertex (or apex), which has been found earlier to be nasally displaced [23], has been similarly located on the nasal side of the geometric center of the cornea as illustrated in Fig. 7. Further, statistically significant correlation was found between y coordinate (vertical direction) of MCT point and that of the geometric center (r = 0.463, P<0.000) and α rotational displacement (r = 0.094, P = 0.014, the coordinate convention has positive α toward the inferior side). While the convergence described above affects the locations of the geometric centers of both anterior and posterior surfaces, the posterior center was further affected by the fact that the thinnest point on the cornea is located away from the apex and in the inferior-temporal region [24], [25].

The general quadratic model, which considers both translational and rotational displacements, was employed in this study to assist in assessment of bilateral cornea symmetry. While it is recognized that more sophisticated geometric models exist such as Zernike [26] and rational functions [27], which have the potential to more accurately represent corneal surface, these models have several limitations. Models based on Zernike polynomials may oscillate between values to be fitted, have poor asymptotic properties [28] and it is hard to assess a priori how many terms are necessary to achieve an acceptable fitting accuracy [29]. Further, nonlinear methods based on rational functions have a relatively simple form and can accommodate a wide range of shapes, but their mathematical properties are not well understood and it can be difficult to select the degrees of numerator and denominator that would lead to the best surface fit [28]. The quadratic model was selected as it could provide accurate estimates of important corneal shape parameters such as curvature and shape factor [30] and a high-quality fit with the topography data.

The translational and rotational displacements (x_0_, y_0_, z_0_, α, β, γ) included in the general quadratic equation are thought to have four sources; (1) the optical distortion caused by aberrations in the Pentacam's measuring lens, (2) the reduced accuracy of data collection in peripheral cornea, (3) the physical translational and rotational misalignments of the eye relative to the instrument, and (4) the geometric imperfections in corneal shape. While it is difficult to separate the effects of the four sources, it can be assumed that the optical distortion of the instrument is small and has a relatively small effect, and that the reduced accuracy in the periphery affects the map edges equally and hence has little influence on the calculations. Further as any eye movement is detected by a second camera and corrected for in the Pentacam system, the mean values of parameters x_0_, y_0_, z_0_, α, β, γ would likely be mainly related to the geometric imperfections of the cornea. This argument could then explain the differences in angles α, β, γ determined for the anterior and posterior surfaces, for which geometric imperfections may differ.

Organization of stromal collagen plays an important role in determining corneal shape and an earlier X-ray scattering study demonstrated that the distribution of preferentially aligned fibrils in the cornea exhibited a degree of midline symmetry between left and right eyes [31]. In this study, we evaluated the notion of midline symmetry in bilateral corneal topography. Although the in vivo topography maps included in the study had wide ranges of refractive error, corneal curvature, astigmatism and thickness, there was a high degree of interocular mirror symmetry between bilateral corneas. Clinically, this finding may be significant as the expected symmetry could provide useful validation of binocular data so that absence of high symmetry in topography may warrant repeat of clinical measurement. An additional clinical application could be in intraocular power calculations for post refractive surgery procedures if only one eye was treated and preoperative data was not available. The existence or otherwise of bilateral mirror symmetry also has important implications for the statistical analysis of corneal biometric parameters as a vector transformation may be necessary if bilateral corneas from the same individual were integrated together in topographic vector analysis.
